# P02.07. A study to assess the validity of applied kinesiology (AK) as a diagnostic tool and as a nonlocal proximity effect

**DOI:** 10.1186/1472-6882-12-S1-P63

**Published:** 2012-06-12

**Authors:** S Schwartz, J Utts, S Spottiswoode, C Shade, L Tully, W Morris, G Nachman

**Affiliations:** 1 Samueli Institute, Langley, USA; 2 Professor, Department of Statistics, University of California – Irvine, Irvine, USA; 3 Managing Director and Chief Scientist, The Resource Group, Beverly Hills, USA; 4 Director of Int. Scientific Advisory Board, Applied Biophysics Found., Boulder, USA; 5 Chief Scientist, Quicksilver Scientific, Boulder, USA; 6 Professor of Osteopathic Manipulation Medicine, A.T. Still Univ., Mesa, USA; 7 Medical Writing Scientist, Quintiles Transnational Corporation, Raleigh-Durham, USA

## Purpose

1. Is there a difference in muscular strength when an individual holds a substance inimical to life processes compared to a substance essential for life? 2. Does the effect involve input from the person being measured, and the kinesiologist doing the measurement, or only the person measured? 3. Is the result the same when different kinesiologists take measurements, or when no kinesiologist is involved? 4. Does belief, expectation, gender, or time perception play a role?

## Methods

Fifty-one participants each participated in 3 trials: a woman kinesiologist, a male kinesiologist, and no kinesiologist with testing using a hand dynamometer. The woman kinesiologist was replaced by another woman part way through study. Results were examined across all three and by practitioner. Each trial used unique pairs of visually identical, randomly numbered, sealed vials; one vial in each pair contained saline solution; the other contained saline solution plus ionic hydroxylamine hydrochloride (NH_3_OH)+. Both trial vials were independently muscle tested using accepted AK techniques. All present were blind to which vial contained ionic hydroxylamine. Kinesiologist force was measured via a pressure pad system. Practitioner and patient independently designated by number the vial in each trial producing the weak response. In the dynamometer trial, muscle weakness was assessed by hand strength.

## Results

Of the 151 sets of trials, the hydroxylamine vial was identified correctly in 80 of them (53%), resulting in a one-tailed exact binomial p-value of 0.258. Results for two of the kinesiologists were almost exactly at chance. For the third kinesiologist there was a one-tailed exact binomial p-value of 0.18 (unadjusted for multiple testing). Results for the dynamometer were also almost exactly at chance. Testing whether there was a significant difference in proportions for whom the AK test worked, based on a belief about whether it would work, resulted in non-significant chi-square values of 0.6 (p = 0.44) for the trials with one kinesiologist, and 2.222 (p = 0.14) for the hand dynamometer trials. The final variable examined was gender. While there was no significant difference in performance for males or the hand dynamometer, the combined data for the two female kinesiologists did reveal a difference. Of the 33 sessions with females, only 15 were successful (45%) while for the 18 sessions with males, 14 were successful (78%) resulting in a chi-square statistic of 4.96, p = 0.026.

## Conclusion

Study and review of the AK literature using QUADAS, STARD, JADAD and CONSORT suggest that research published by the AK field fails accepted scientific standards. AK in this study fails as a reliable diagnostic tool upon which health decisions could be based.

